# Predominance of low pathogenic avian influenza virus H9N2 in the respiratory co-infections in broilers in Tunisia: a longitudinal field study, 2018–2020

**DOI:** 10.1186/s13567-023-01204-7

**Published:** 2023-10-03

**Authors:** Adam Jbenyeni, Guillaume Croville, Christophe Cazaban, Jean-Luc Guérin

**Affiliations:** 1 IHAP, Université de Toulouse, INRAE, ENVT, Toulouse, France; 2 Ceva Santé Animale S.A., Libourne, France

**Keywords:** Mixed infections, poultry, respiratory disease complex, influenza A Virus, H9N2 LPAIV, IBV, aMPV, NDV

## Abstract

Respiratory diseases are a health and economic concern for poultry production worldwide. Given global economic exchanges and migratory bird flyways, respiratory viruses are likely to emerge continuously in new territories. The primary aim of this study was to investigate the major pathogens involved in respiratory disease in Tunisian broiler poultry and their epidemiology. Between 2018 and 2020, broilers farms in northeastern Tunisia were monitored, and 39 clinically diseased flocks were sampled. Samples were screened for five viral and three bacterial respiratory pathogens using a panel of real-time PCR assays. The reemergence of H9N2 low pathogenic avian influenza virus (LPAIV) in commercial poultry was reported, and the Northern and Western African GI lineage strain was typed. The infectious bronchitis virus (IBV) GI-23 lineage and the avian metapneumovirus (aMPV) subtype B also were detected for the first time in broilers in Tunisia. H9N2 LPAIV was the most detected pathogen in the flocks tested, but rarely alone, as 15 of the 16 H9N2 positive flocks were co-infected. Except for infectious laryngotracheitis virus (ILTV), all of the targeted pathogens were detected, and in 61% of the respiratory disease cases, a combination of pathogens was identified. The major combinations were H9N2 + aMPV (8/39) and H9N2 + IBV (6/39), showing the high contribution of H9N2 LPAIV to the multifactorial respiratory diseases. This field survey provided evidence of the emergence of new respiratory viruses and the complexity of respiratory disease in Tunisia. A comprehensive and continuous surveillance strategy therefore is needed to better control respiratory pathogens in Tunisia.

**Supplementary Information:**

The online version contains supplementary material available at 10.1186/s13567-023-01204-7.

## Introduction

Over the past 20 years, the commercial poultry sector has grown rapidly in Tunisia. Production levels have doubled, and poultry have become the main source of protein in the country (FAO). Respiratory diseases pose a major health threat to commercial poultry worldwide, causing tremendous economic losses. The H9N2 low pathogenic avian influenza virus (LPAIV) is the most prevalent avian influenza virus in the world [1]. In the late 1990s, H9N2 LPAIV already had been detected in Southeast Asia and the Middle East in domestic poultry. Since the 2000s, H9N2 LPAIV has become enzootic in Asia, the Middle East, and North and West Africa [2].

H9N2 LPAIV was detected in Tunisia for the first time in 2009. The strain, assumed to have originated from Pakistan, was replaced in 2012 by a genetically related virus that originated from the United Arab Emirates (UAE) and spread to Tunisia through Libya [3–5]. In early 2018, the surveillance of H9N2 LPAIV in migratory birds in Tunisia enabled the detection of two different strains of H9N2 LPAIV from the same lagoon in the northeast region of the country. The first was closely related to the Tunisian H9N2 strain of 2012 and was isolated from wild birds, whereas the second fell in the Northern and Western African H9N2 cluster and was isolated from lagoon water [6]. The earliest virus in the latter cluster was detected in Morocco in 2016, and has since become endemic in several North and West African countries like Algeria, Burkina-Faso, Ghana, Togo and Benin [5, 7–10]. An H9N2 virus from the same cluster was detected in an infant in Senegal in 2019, exhibiting its potential zoonotic risk [11]. Over the past decade, other respiratory viruses affecting poultry also have emerged in Tunisia, including Newcastle disease virus (NDV) genotype VII.2 in 2013, a novel variant of infectious bronchitis virus (IBV) in 2016, and infectious laryngotracheitis virus (ILTV) between 2013 and 2016 [12, 13].

Previous studies have provided evidence of the multifactorial origin of respiratory diseases. A primary infection can be complicated by environmental factors (ammonia, dust, moisture etc.) and/or by co-infection with other pathogens [14–16]. Co-infection is likely to enhance the clinical signs and mortality of each of the pathogens involved through synergistic mechanisms operating between them. This seems to be true for H9N2 LPAIV, which under experimental conditions barely induces clinical signs in chickens, while in the field is correlated with severe clinical signs and high mortality [10, 17]. Several experimental infections succeeded in reproducing H9N2 LPAIV clinical signs in the field by co-infecting H9N2 alongside another pathogen such as IBV, NDV or *E. coli* [18–21].

Given the repeated emergence and circulation of respiratory pathogens in the Tunisian commercial poultry sector, a comprehensive and longitudinal surveillance study is needed. In this study, we monitored broiler flocks in northeast Tunisia over 3 years to investigate the pathogens involved in the respiratory disease outbreaks observed and assess their longitudinal circulation. In the end we brought to light the emergence of three respiratory viruses on broiler farms in Tunisia between 2018 and 2020. We also conveyed that the respiratory diseases have a multifactorial etiology as 2/3rd of the flocks tested were co-infected.

## Materials and methods

### Context and sampling protocol

**Figure 1:**
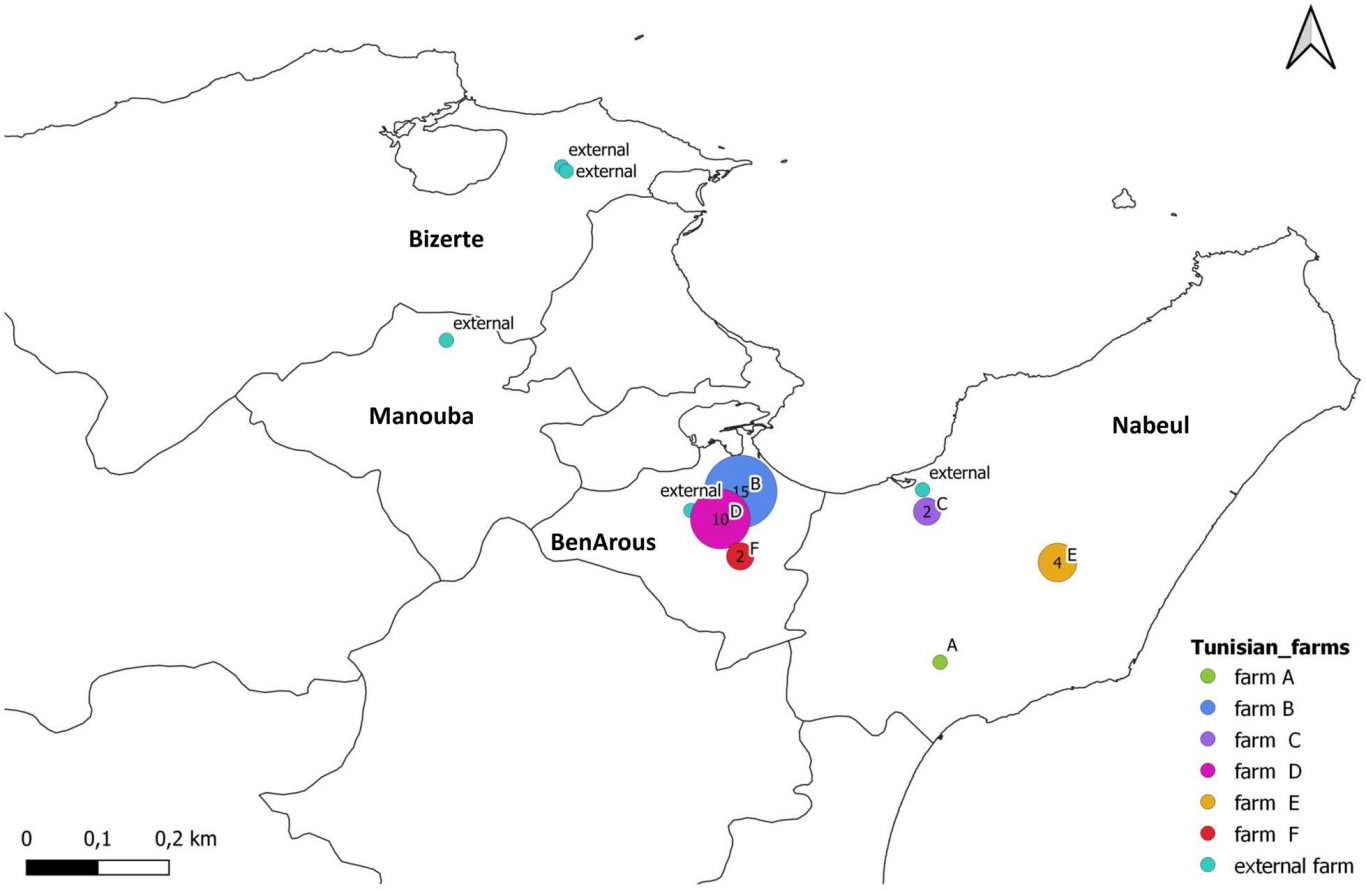
**Geographic distribution of tested flocks on monitored and external broiler farms in Tunisia.** Circles on the map represents the surveyed farms and the numerical value represents the cumulative number of sampled flocks in each farm. The monitored farms were distributed in two governorates, Nabeul in the east of the country, and Ben Arous in the west. The map was created using QGIS3 software

From January 2018 to June 2020, six farms belonging to the same broiler rearing company in Tunisia were monitored for respiratory outbreaks. These farms were located either in Ben Arous or in Nabeul, two northeastern Tunisian governorates (Figure 1). The farms were named using letters from A to F. Flocks showing acute respiratory signs were sampled within the first 5 days of an outbreak. The number of flocks sampled from the same farm during an outbreak depended on the health status of each one of the flocks. For each flock, 12 tracheal swabs were collected from diseased or freshly dead birds and immediately smeared onto the four circles of an FTA^®^ card, with three swabs per circle. FTA^®^ cards were dried for at least five minutes, labelled, placed in a Ziploc bag and transported on ice to be stored at −20 °C until later use [22]. A written form including information about the GPS location, age, date of the first respiratory signs, daily mortality and vaccination history was provided with each sampled flock.

### FTA card preparation and nucleic acid extraction

Three disks of 2 mm diameter were punched out of each FTA^®^ card’s circle using a Whatman Harris Uni-Core^™^ puncher and pooled together in a 2 mL microcentrifuge tube with 300 µL of TE Buffer (10 mM Tris, 1 mM EDTA, pH8.0) to soak the disks. The puncher was cleaned using pure ethanol between circles and cards. For nucleic acid elution, microcentrifuge tubes were vortexed for two hours with Vortex-Genie^®^ 2 then briefly centrifuged. Then 150 µL of eluent were used for RNA and DNA extraction using the NucleoSpin RNA Virus extraction kit (Macherey–Nagel, Düren, Germany) following the manufacturer’s instructions.

### Screening of respiratory pathogens

Samples were screened for a panel of eight respiratory pathogens including H9N2 LPAIV, IBV, NDV, aMPV and ILTV, *Mycoplasma gallisepticum* (MG), *Mycoplasma synoviae* (MS), and *Ornithobacterium rhinotracheale* (ORT). Primers used for this study are listed in Additional file 1 [23-30]. Depending on the different pathogens’ nucleic acid class, real-time PCR (qPCR) or RT-real-time PCR (RT-qPCR) assays were performed on an Agilent Bravo Automated Liquid Handling Platform (Santa Clara, USA) and an Applied Biosystems ViiA7 thermocycler (Foster City, USA).

### RT-qPCR for RNA amplification

The RNA viruses were screened using the iTaq^™^ universal SYBR^®^ green one-step RT-qPCR kit (Bio-Rad, Hercules, USA). The reaction mix consisted of 5 µL of 2 × iTaq mix, 0.125 µL of iScript reverse transcriptase, 0.3 µL of each primer (10 µM), 2.275 µL of nuclease free water and 2 µL of RNA with the following program: reverse transcription at 50 °C for 10 min and polymerase activation at 95 °C for 1 min followed by 35 cycles of denaturation at 95 °C for 15 s and annealing/extension at 60 °C for 60 s. AIV M gene positive samples were further subtyped using [31] H9 primers following the same reaction setup and thermal cycling conditions of one-step RT-qPCR on a LightCycler 96^®^ system (Roche Applied Science, Penzberg, Germany).

### qPCR for DNA amplification

The DNA viruses and bacteria were screened using LightCycler^®^ 480 SYBR Green I Master qPCR kit (Roche Applied Science, Mannheim, Germany). The reaction mix consisted of 5 µL of Master mix, 0.2 µL of each primer (10 µM), 2.6 µL of nuclease free water and 2 µL of DNA with the following program: pre-incubation at 95 °C for 10 min followed by 45 cycles of denaturation at 95 °C for 10 s, annealing at 60 °C for 15 s and extension at 72 °C for 15 s.

### Sanger sequencing

Sequencing was performed only on material from FTA cards. The HA gene of H9N2 LPAIV was sequenced using universal primers described by Hoffmann et al. [32]. The complete fusion gene of NDV was sequenced using Esmaelizad et al. [33] primers to differentiate vaccine type strains from field challenge strains. A partial sequence (~950 base pairs) overlapping the small hydrophobic (SH) and attachment (G) gene of aMPV was generated using SH1 + and G6- primers described by Cecchinato et al. [34]. The partial S1 gene sequence of IBV (~300 base pairs) spanning the hypervariable region 3 (HVR3) was generated using Worthington et al. [35] nested PCR. For all viruses, PCR products were loaded on agarose gel and DNA fragments with the expected length were excised and purified using NucleoSpin^®^ Gel and PCR Clean-up kit (Macherey–Nagel, Düren, Germany) according to the supplier’s instructions. Purified DNA was prepared for Sanger sequencing and shipped to the sequencing external service of Eurofins genomics (Cologne, Germany).

### Sequence treatment and phylogenetic analysis

Fasta format sequences were manually treated using the BioEdit v7.2.5 software package and consensus sequences were generated. The BLAST program [36] was used for comparison with sequences in the NCBI database. Sequences with high identity percentages were used to construct phylogenetic trees. MAFFT multiple sequence alignment program online version 7 was used to align sequences [37]. Maximum likelihood phylogenetic trees with 1000 bootstraps were created using MEGA 10 software: Molecular Evolutionary Genetics Analysis version 10.

## Results

### Farms and flocks

During the study period, a total of 39 flocks were investigated and sampled between January 2018 and June 2020. Due to logistical reasons, monitoring and sampling activities were interrupted between April and October 2019. Of these 39 flocks, 34 originated from the six monitored farms in the Ben Arous and Nabeul governorates (Figure 1). The number of flocks sampled from farms A, B, C, D, E and F were 1, 15, 2, 10, 4 and 2, respectively (Additional file 2). The five additional flocks came from farms that were not affiliated with the rearing company (referred to as “external farms”) (Additional file 2) and were located in the Ben Arous, Nabeul, Bizerte and Manouba governorates (Figure 1). The median age of all 39 flocks studied was 31 days, with a minimum age of 15 days. The daily mortality of the flocks studied ranged from 0.05 to 14.36% during the outbreak (Additional file 3). The respiratory signs observed included sneezing, coughing, foamy eye, and dyspnea. In terms of lesions, tracheitis, fibrinous cast in the trachea, pneumonia, petechial proventriculitis and inflammation of Harderian gland, spleen and caeca tonsils were recorded. All flocks were vaccinated against IBV and NDV. The IBV vaccination schedule comprised priming at the hatchery with a Mass-like vaccine and boosting on the farm with a 793B-like vaccine. For NDV, birds were primed at the hatchery with either a combination of a recombinant and a live virus vaccine or with a live virus vaccine only, and were boosted every 10 days using a live virus vaccine. None of the flocks had been vaccinated for H9N2 LPAIV or aMPV.

### Molecular epidemiology

The qPCR assays showed a high detection of viral pathogens with at least one of the five screened viruses per flock. For the 39 tested flocks, 56 positive results for viruses were recorded. H9N2 LPAIV, IBV, aMPV and NDV were detected in 21 (54%), 20 (53%), 11 (28%), and 4 (10%) flocks, respectively. ILTV was not detected in any of the flocks. MS, ORT and MG were detected in 13 (33%), 12 (31%) and one (3%) flock, respectively (Table 1).

**Table 1:** **qPCR positiveresults and number of pathogens detected per flock**

Pathogen	Viral				Bacterial			Number of pathogens detected per flock
Flocks ID	H9N2	NDV	IBV	aMPV	Ms	Mg	ORT	
18–001	–^a^	–	1	–	–	–	N.D	1
18–003	–	–	1	–	1	–	N.D	2
18–004	1^b^	–	–	–	1	–	N.D	2
18–007	1	–	–	–	1	–	N.D	2
18–008	1	–	–	–	–	–	N.D	1
18–010	1	–	–	–	–	–	N.D	1
18–011	–	–	1	–	–	–	N.D	1
18–012	1	–	1	–	–	–	N.D	2
18–013	1	–	1	–	–	–	N.D	2
18–014	1	–	–	–	–	–	N.D	1
18–015	1	–	1	–	–	–	N.D	2
18–016	–	–	1	–	–	–	N.D	1
18–017	1	–	1	–	–	–	N.D	2
18–019	1	–	1	–	–	–	N.D	2
18–020	–	–	1	–	–	–	N.D	1
18–021	–	–	1	–	–	–	N.D	1
18–022	–	–	1	–	–	–	N.D	1
18–023	–	–	1	–	–	–	N.D	1
18–026	–	–	1	–	1	–	N.D	2
18–028	–	–	1	–	–	–	N.D	1
18–029	–	–	1	–	–	–	N.D	1
19–035	–	1	–	–	–	–	N.D	1
19–036	–	1	–	–	1	–	N.D	2
19–037	–	1	–	–	–	–	N.D	1
19–038	–	1	–	–	–	–	N.D	1
19–042	–	–	1	–	–	1	N.D	2
20–047	–	–	1	1	–	–	N.D	2
20–048	–	–	–	1	–	–	1	2
20–049	1	–	–	–	–	–	1	2
20–051	1	–	–	1	–	–	1	3
20–052	1	–	1	–	1	–	1	4
20–053	1	–	–	1	1	–	1	4
20–054	1	–	–	1	1	–	1	4
20–055	1	–	–	1	1	–	1	4
20–056	1	–	–	1	–	–	1	3
20–057	1	–	–	1	1	–	1	4
20–058	1	–	1	1	1	–	1	5
20–059	1	–	–	1	1	–	1	4
20–060	1	–	–	1	1	–	1	4
Total	21	4	20	11	13	1	12	82

In this table, are reported the qPCR positive results and the number of pathogens detected per flock, as well as the total number of detections for the eight screened pathogens: H9N2, low pathogenic avian influenza virus

aMPV: Avian metapneumovirus, IBV: Infectious Bronchitis virus, ILTV: Infectious Laryngotracheitis virus, MG: *Mycoplasma gallisepticum*, MS: *Mycoplasma synoviae*, NDV: Newcastle Disease virus, ORT: *Ornithobacterium rhinotracheale*, N.D: not done

^a^Negative

^b^Positive

Although monitoring began in January 2018, it was not until April 2018 that H9N2 LPAIV was first detected. This virus continued to be detected until the end of the study on all of the monitored farms, exhibiting two waves of outbreaks. IBV was detected throughout the 3 year study on all of the monitored farms except farms A and C. NDV was recorded in two flocks of farm D and in one external flock during the same period in February 2019. aMPV was first detected in February 2020, and continued to be detected until the end of the study on monitored and external farms (Table 1).

### Respiratory co-infections

Altogether, the PCR tests performed on the 39 study flocks showed a total of 82 positive results, covering 4 viruses and 3 bacteria included in the panel, which confirms frequent co-infections and the diversity of respiratory pathogens in these Tunisian poultry holdings. The co-infections were detected in 24 (61%) of the 39 flocks and involved viruses and/or bacteria in combinations of two, three, four or five pathogens in 14, 2, 7 and 1 flock, respectively (Table 1). Co-infections involving only viruses occurred in 16 flocks. These viral-viral co-infection combinations were mainly H9N2 + aMPV, H9N2 + IBV, IBV + aMPV and H9N2 + IBV + aMPV, profiled in 8, 6, 1 and 1 co-infections, respectively. H9N2 LPAIV was the most involved pathogen, found in 15 of the 16 viral co-infected flocks. Co-infections involving viruses and bacteria occurred in 18 of the 24 co-infected flocks, and were divided in two groups. The first consisted of a bacterial superinfection of a viral-viral co-infection and occurred in 10 flocks; the second consisted of a bacterial superinfection of a single virus infection and occurred in 8 flocks. Again, H9N2 LPAIV was the most involved pathogen in the co-infections, with 18/21 H9N2 LPAIV positive flocks. H9N2 LPAIV co-infections were detected with ORT, MS, aMPV and IBV in 11, 10, 9, 7 flocks, respectively.

### Phylogenetic analysis

#### H9N2 LPAIV

**Figure 2:**
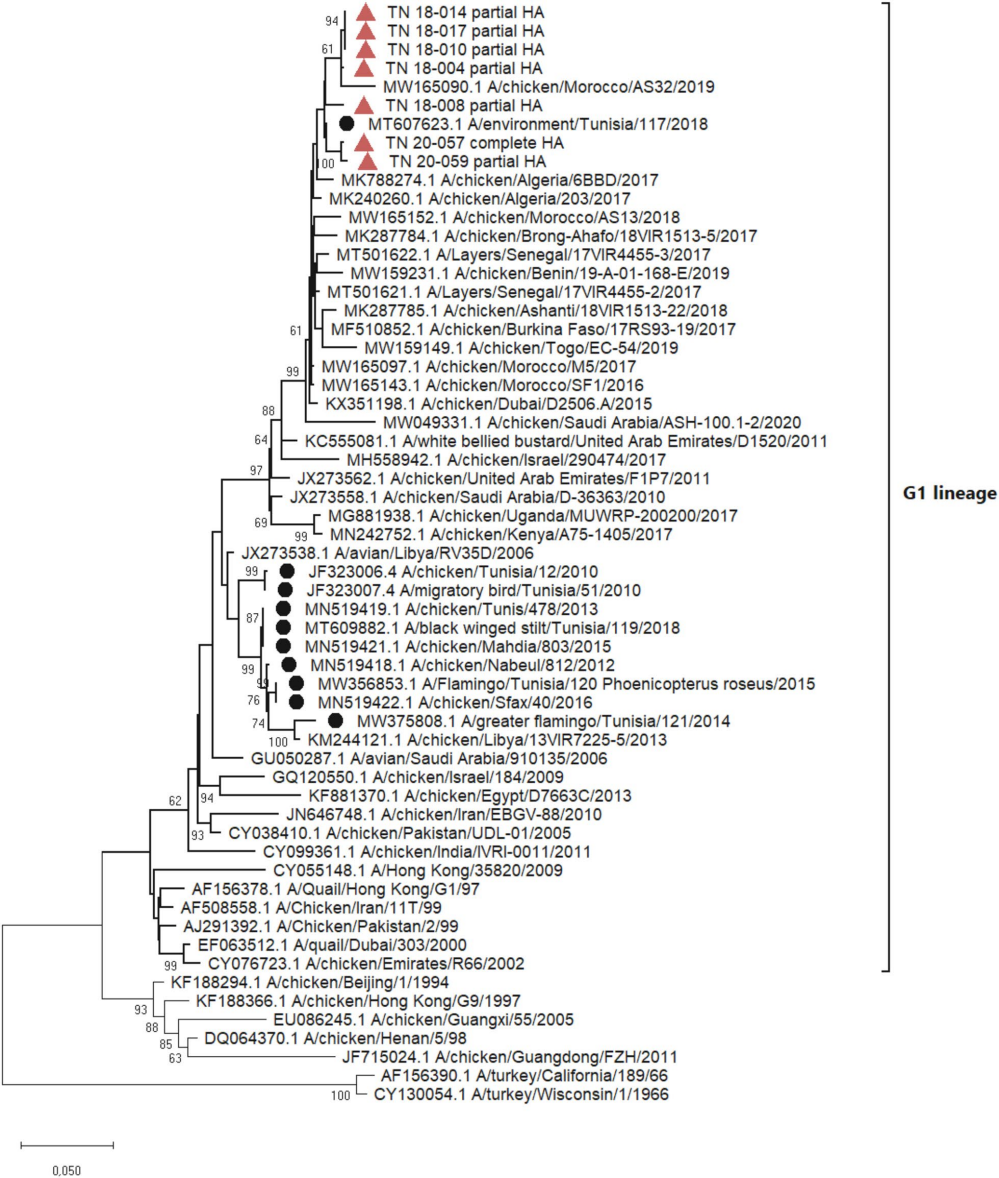
**Phylogenetic tree of H9N2 LPAIV. The tree was constructed using the HA gene nucleotide sequences.** Sequences from this study were labelled with red triangle, Tunisian sequences available in GenBank were labelled with black circle and G1 lineage sequences from Africa and the Middle East and reference sequences of other H9N2 lineages were unlabelled. The evolutionary history was inferred using the Maximum Likelihood method with General Time Reversible model and 1000 bootstrap replications in MEGA X. All nucleotide positions containing gaps and missing data were eliminated. A total of 924 positions were included in the final dataset. Only bootstrap values higher than 60 were conserved

Complete (1.7 kb) or partial (1 kb) HA sequences of H9N2 LPAIVs from seven flocks were generated. HA gene sequences and related metadata were deposited in the GenBank database under accession numbers OQ179924-OQ179930 (Additional file 4). The phylogenetic tree showed that Tunisian H9N2 LPAIV viruses belonged to the h9.4.2 clade of the G1-like lineage and have a common ancestor with the United Arab Emirates H9N2 LPAIV virus (JX273562.1) (Figure 2). Our Tunisian H9N2 viruses clustered with those detected in Algeria and Morocco. The H9N2 LPAIV viruses detected during this study exhibit a nucleotide pairwise distance of 10% from the Tunisian H9N2 LPAIV of 2010.

#### Other viruses

##### NDV

**Figure 3:**
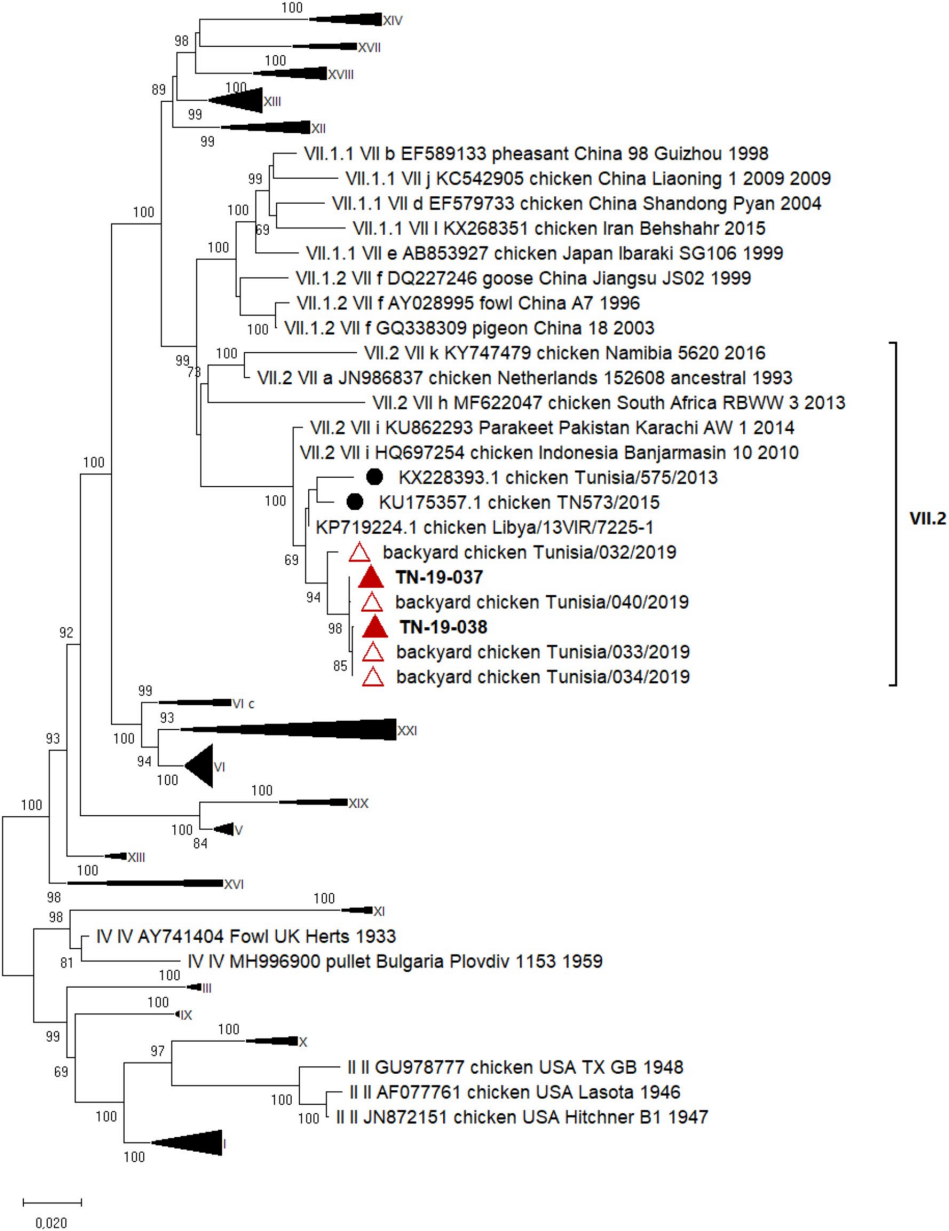
**Phylogenetic tree of NDV. The tree was constructed using F gene nucleotide sequences of Class II NDV.** Sequences from this study were labelled with filled red triangle, previous NDV Tunisian sequences available in GenBank were labelled with black circle, 2019 NDV sequences from Tunisian backyard chickens were labelled with empty red triangle and reference sequences were unlabelled. The evolutionary history was inferred using the Maximum Likelihood method with Tamura 3-parameter model and 1000 bootstrap replications in MEGA X. All nucleotide positions containing gaps and missing data were eliminated. A total of 1675 positions were included in the final dataset. Only bootstrap values higher than 60 were conserved

Complete F gene sequences of NDVs from one monitored flock and one external flock were generated. F gene sequences and related metadata were deposited in the GenBank database under accession numbers OQ199524 and OQ199525 (Additional file 4). Referring to the revised nomenclature recommended by [38], the phylogenetic analysis showed that NDV sequences belonged to the genotype VII.2 (formerly VII-i) and shared 97% of nucleotide identity with the 2013 and 2015 Tunisian genotype VII.2 strains (Figure 3).

##### aMPV

**Figure 4:**
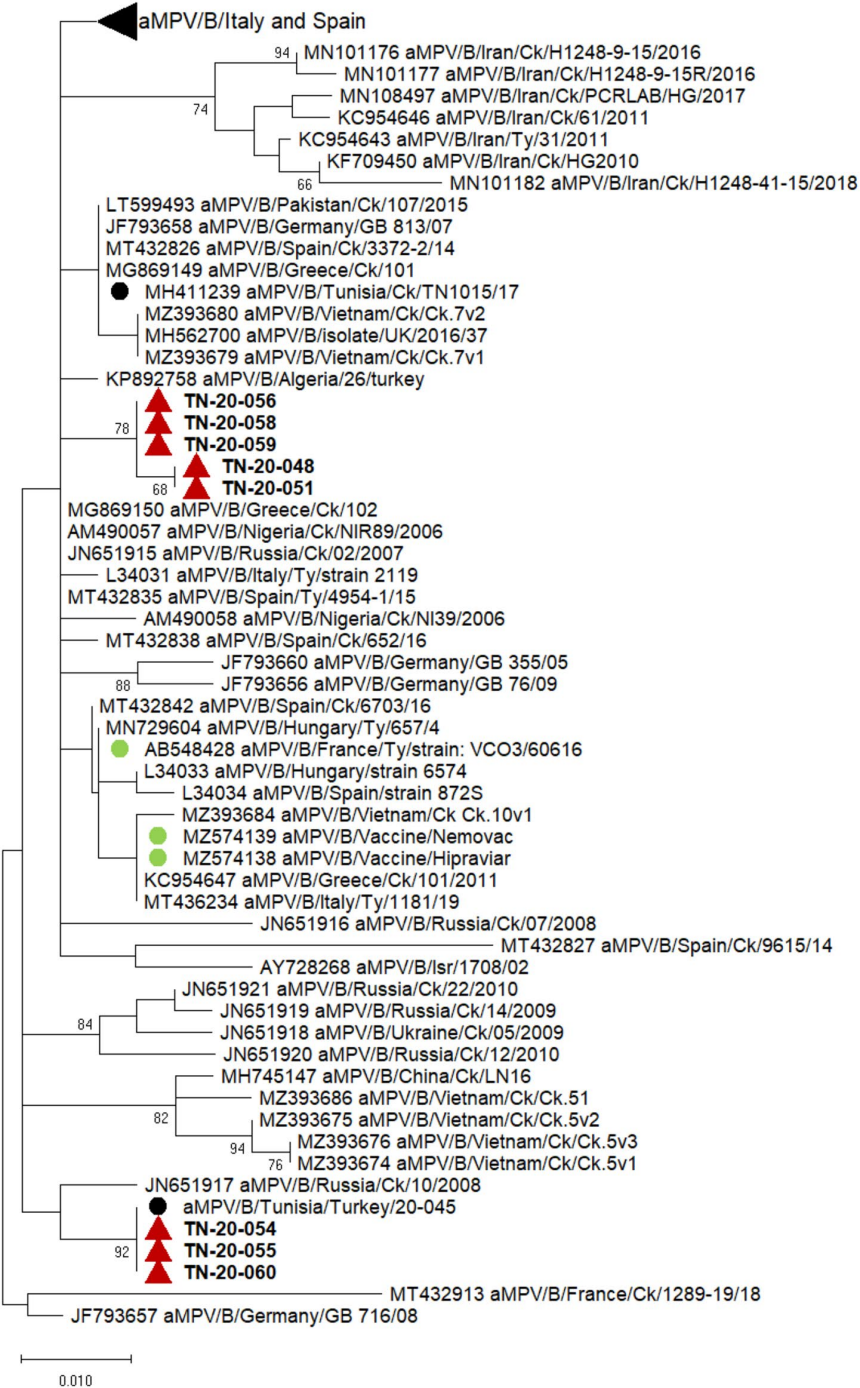
**Phylogenetic tree of aMPV.** The tree was constructed using G gene nucleotide sequences. Sequences from the present study were labelled with red triangle, previous Tunisian sequence were labelled with black circle, vaccine sequences were labelled with green circle and other aMPV-B sequences available in GenBank were unlabelled. The evolutionary history was inferred using the Maximum Likelihood method with Tamura 3-parameter model and 1000 bootstrap replications in MEGA X. All nucleotide positions containing gaps and missing data were eliminated. A total of 292 positions were included in the final dataset. Only bootstrap values higher than 65 were conserved

**Figure 5:**
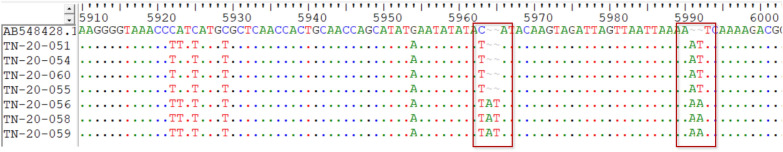
**The genetic polymorphic features of Tunisian aMPV sequences.** The alignment of the noncoding region between SH and G genes of Tunisian aMPV sequences from this study with VCO3 reference sequence showed the presence of two motifs of nucleotide insertions highlighted in the red frames

Partial SH and G gene sequences of aMPVs were generated from eight flocks. G gene sequences and related metadata were deposited in the GenBank database under accession numbers OQ199508 and OQ199515 (Additional file 4). The phylogenetic analysis sorted G gene sequences into two different clusters within the subtype B, and were distinct from aMPV vaccine strains (Figure 4). The alignment of the SH and G gene sequences of the detected aMPVs and the reference sequence of aMPV revealed some genetic polymorphic features. These features consist of two or four nucleotide insertions in the noncoding region between the SH and G genes. Based on these two features, we were able to retrieve the two clusters obtained by the phylogenetic analysis of the G gene sequences. Two motifs of nucleotide insertions were found; the first involved the insertion of two nucleotides and the second the insertion of four nucleotides (Figure 5).

##### IBV

**Figure 6:**
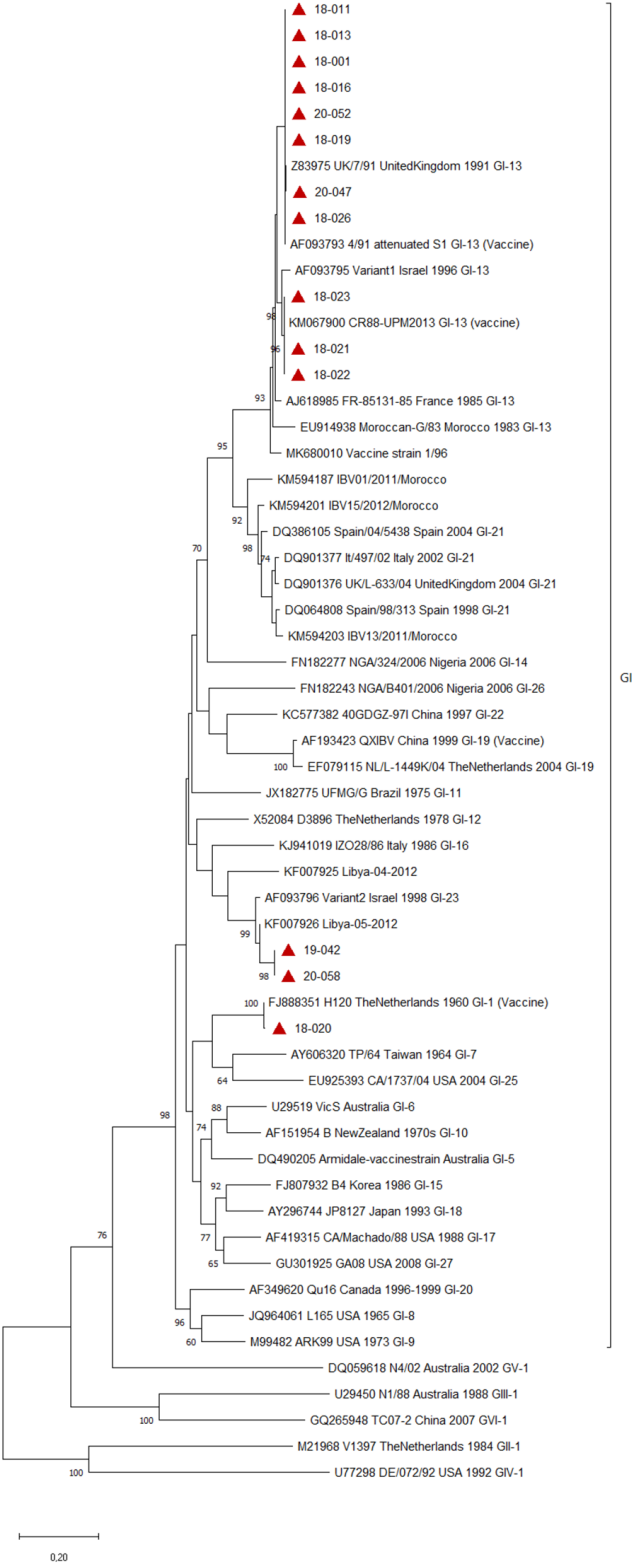
**Phylogenetic tree of IBV. The tree was constructed using IBV HVR3 nucleotide sequences.** The sequences from the present study were labelled with red triangle and reference sequences of the different IBV lineages available in GenBank were unlabelled. The evolutionary history was inferred using the Maximum Likelihood method with General Time Reversible model and 1000 bootstrap replications in MEGA X. A total of 1875 positions were included in the final dataset. Only bootstrap values higher than 60 were conserved

The partial S1 gene sequence of IBV was generated from 13 flocks. These sequences and related metadata were deposited in the GenBank database under accession numbers OQ199494 and OQ199507 (Additional file 4). To distinguish between vaccine and field type viruses, nucleotide identity between the detected IBV sequences and IBV vaccine sequences was used. The detected IBVs were considered a vaccine type when their nucleotide identity with a vaccine strain was 99% or more, and were considered a field challenge when their nucleotide identity was less than 99% [35]. Adopting [39] nomenclature, the phylogenetic analysis showed the presence of three GI lineages. Eleven, two and one sequence respectively fell in the GI-13, GI-23 and GI-1 lineages. GI-13 were 793B vaccine-like sequences and homologous to either 4/91 (eight sequences) or CR88 vaccine strains (three sequences). The GI-23 sequences shared 95% of nucleotide identity with both the Israeli variant 2 strain (AF093796) and the Libyan GI-23 strains detected in 2012 (Figure 6).

## Discussion

In this study, we monitored over a 3 year-period spontaneous acute respiratory cases on broiler farms operated by the same rearing company in Tunisia. Our aim was to investigate the major respiratory pathogens involved in respiratory diseases in the northeastern region of Tunisia and their longitudinal circulation.

The design of our study, which involved targeting a large panel of respiratory pathogens and sampling clinically diseased birds during the early stage of a respiratory outbreak, enabled us to detect at least one viral pathogen per flock with high viral loads. We even detected aMPV (Ct 15), which is known to be difficult to detect due to its short shedding window (Additional file 3) [40].

Importantly, we found that H9N2 LPAIV was the most detected respiratory pathogen (54%) on the farms monitored. H9N2 LPAIV was enzootic on the monitored farms in 2018 and 2020, with outbreaks occurring during spring and summer. However, we cannot confirm the seasonality of H9N2 LPAIV due to the interruption of field monitoring that occurred during the study period. Further investigation is therefore required. The phylogenetic analysis revealed that the H9N2 LPAIV viruses detected in 2018 and 2020 were monophyletic, belonged to the G1 lineage and clustered with Northern and Western African viruses introduced from the Middle East in 2016 [5]. In addition, the H9N2 LPAIV strain reported here was distant from the old Tunisian G1 lineage that emerged in 2009 and that was detected until 2016 on poultry farms, and until 2018 in migratory birds [3, 4]. The phylogenetics of H9N2 LPAIV suggest a new introduction of H9N2 LPAIV in Tunisia. This study is the first to report the circulation of the Northern and Western African clade of H9N2 LPAIV in poultry in Tunisia. Interestingly, during the same period in 2018, the first detection of H9N2 LPAIV coincided with the detection of a very closely related virus (99%) from the water of a lagoon in the same region of Tunisia [6]. Our findings support the hypothesis proposed by Larbi et al. [6] that the transmission of H9N2 LPAIV occurs from poultry farms to migratory birds via their contamination of the environment as strains isolated from the water and the migratory birds on the same site were different. Considering the close phylogenetic relatedness between the H9N2 LPAIV introduced in Tunisia and those circulating in neighboring countries during the same period, we assume that the new introduction occurred through the trade of poultry or poultry products.

The second most detected virus on the farms monitored was IBV (53%), with an enzootic circulation over the study period. The phylogenetic analysis showed the dominance of GI-13 vaccine-like strains (793B). This finding is common as GI-13 live virus vaccines are used widely on poultry. The rolling infection of birds with vaccine viruses within a flock, when the initial coverage is incomplete, can lead to vaccination reactions, thus complicating diagnostic efforts [41]. Given the epidemiological context in Tunisia, an infection with a rolling IBV vaccine strain could be co-infected by H9N2 LPAIV which leads to the exacerbation of the clinical signs [42, 43]. In this study, we report the detection of the GI-23 lineage (variant 2) for the first time in Tunisia. Over the 3 years of the study period, variant 2 was detected twice, 5 months apart, on the same farm. The lack of spread of the GI-23 virus to the other farms monitored and its occasional detection suggest a cross protection of Massachusetts and 793B combination against variant 2, as also described in previous studies [44, 45]. However, we cannot draw conclusions about the prevalence of this lineage in Tunisia; active and continuous surveillance of this variant therefore is required.

The detection of aMPV started in February 2020 and lasted until June 2020. During this period, aMPV was detected on both monitored and external farms (28%) in the different governorates, indicating the emergence and circulation of a new aMPV in broilers. Lachheb et al. [46] also reported the detection of an aMPV field strain in 2019 in broilers. Unfortunately, we were not able to determine the phylogenetic relatedness of the previously described virus with the detected aMPVs during this study as the sequence was not published. The phylogenetic analysis grouped the detected aMPVs in two different clusters within the subtype B and outside of the vaccine strains cluster, suggesting the circulation of two different field strains. We noticed that these aMPVs held polymorphic features in their SH-G non-coding region which do not exist in other aMPVs. Noncoding regions have different biological roles, including the regulation of viral replication, viral persistence, host immune evasion, and cellular transformation [47]. These insertions consequently should not be neglected, and pathogenicity studies are required.

NDV genotype VII.2 was detected during the same period in two flocks on the same monitored farm and on one external farm, showing an epizootic circulation of this virus. This sporadic detection of NDV could be explained by the reinforcement of NDV control measures. After genotype VII.2 emerged in 2013 in Tunisia, it was recommended to add NDV live vaccine boosters every 10 days in broilers and to use NDV vector vaccines. The phylogenetic analysis of NDV showed that the detected viruses are identical and share the most recent common ancestor with previously reported viruses in Tunisian (KU175357) and Libya [48].

Bacterial pathogens also were highly detected, even though samples were collected in the acute respiratory phase showing an early bacterial superinfection. To the best of our knowledge, mycoplasma prevalence in poultry in Tunisia has not yet been studied. Based on the global prevalence of MG and MS, the low detection of MG (one flock) in contrast with the high detection of MS (33%) is in line with their reported occurrence in all poultry populations. However, our results are not in line with the global occurrence of MG and MS in broiler poultry, which are reported to be approximately the same (around 25%) [49]. The lower rate of MG compared to MS could be explained by control measures that are oriented more toward MG than MS in breeding birds, thereby reducing MG vertical transmission in broilers. Considering these results, we are tempted to say that control measures in Tunisia have reduced the occurrence of MG on broiler farms. Nevertheless, more studies on mycoplasma in different poultry populations are needed to monitor the effectiveness of MG and MS control measures and adapt them accordingly. Surprisingly, the detection rate of ORT was very high (100%) in the 12 flocks tested. The screening of ORT was included in our respiratory panel in 2020 and only flocks sampled that year were tested. Even so, these results draw our attention to the likely contribution of this pathogen to respiratory disease in broilers. Our results are consistent with the few existing ORT studies in other countries suggesting the underdiagnosis of this pathogen [21, 50–52].

Interestingly, co-infections between respiratory pathogens were very frequent as 61% of the flocks studied were positive for two or more pathogens. Our results are consistent with the results of similar studies [15, 16] confirming the complexity of respiratory disease and its diagnosis in the field. Viral co-infections occurred in 16 of the 39 flocks studied; of these, H9N2 LPAIV was the major contributor (15/16). The dominance of co-infections by H9N2 LPAIV could be explained by the high detection rate of H9N2 LPAIV in the flocks studied, and could explain the clinical signs associated with H9N2 LPAIV positive flocks. In these co-infections, the major viral combinations were H9N2 + IBV (6/16) and H9N2 + aMPV (8/16). Several experimental studies have shown that co-infections with H9N2 LPAIV and IBV consistently result in more severe clinical signs than single infections with either H9N2 LPAIV or IBV, regardless of the virulence of the IBV strain [17, 42, 43]. This synergy between H9N2 LPAIV and IBV could be explained either by the severe inflammatory response induced by IBV [20], or by the trypsin-like serine protease encoded by IBV which probably facilitates the cleavage activation of the hemagglutinin of the H9N2 LPAIV virus, and thus increases its replication [53, 54]. In contrast, little is known about co-infection mechanisms between H9N2 LPAIV and aMPV as to the best of our knowledge this co-infection has not been previously reported. Clinical manifestations of aMPV in broilers are absent or mild, so co-infections with H9N2 LPAIV could explain the clinical manifestation of flocks positive for aMPV [55]. All of the flocks co-infected with H9N2 and aMPV also were positive for ORT, making it even more difficult to understand the sequential order of co-infections and the synergistic effect between the three pathogens. Only dual co-infections between ORT and H9N2 or aMPV and ORT were previously studied by experimental infections. Pan et al. [21] showed that in broilers, a co-infection involving H9N2 and ORT, either simultaneously or primed by ORT, increased mortality by 60–70% respectively comparing to a single infection with H9N2 LPAIV. Similarly, Marien et al. [56] showed that a prior infection of ORT with aMPV enabled the reproduction of respiratory disease in specific pathogen free (SPF) turkeys, which was not possible by the inoculation of ORT alone via the natural infection route.

In conclusion, this study brought to light the emergence of three new respiratory viruses in Tunisia, as well as several respiratory co-infections. The introduction of a new G1 lineage of H9N2 LPAIV, GI-23 lineage of IBV and subtype B of aMPV was shown, which highlights the importance of active and continuous surveillance and points to an urgent need to adjust control measures to the current situation. The high frequency of the co-infection between H9N2 LPAIV and aMPV suggests that it would be relevant to study the mechanisms of a likely synergism between these two viruses. The complexity of respiratory disease demonstrated in the study emphasizes the need to adopt a comprehensive diagnostic approach for multifactorial respiratory diseases.

### Supplementary Information

**Additional file 1: Oligonucleotides set used for the PCR screening of respiratory pathogens.** The targeted gene, the primers’ nucleotide sequences and the amplicon size for each one of the eight screened pathogens were listed in this file.

**Additional file 2: Investigated flocks’ information.** The date of sampling, the farm and governorate of origin, the size and the age of the investigated flocks were showed in this file.

**Additional file 3: The qPCR Ct values of detected pathogens.** The Age, the mortality, and the qPCR Ct values of the detected pathogens per flocks were listed in this file.

**Additional file 4: GenBank accession numbers of nucleotide sequences generated during this study.** Virus sequences generated in this study were submitted to GenBank database under the accession numbers listed in this additional file.

## Data Availability

The datasets used and/or analysed during the current study are available from the corresponding author upon reasonable request. The dataset supporting the conclusions of this article is included within the article.
