# Supplementary material for: Predominance of low pathogenic avian influenza virus H9N2 in the respiratory co-infections in broilers in Tunisia: a longitudinal field study, 2018–2020
Source: Vet Res. 2023 Oct 3;54:88. doi: 10.1186/s13567-023-01204-7 (PMC10548753; doi:10.1186/s13567-023-01204-7)
Supplement: Supplementary file 1 — Additional file 1: Oligonucleotides set used for the PCR screening of respiratory pathogens. The targeted gene, the primers’ nucleotide sequences and the amplicon size for each one of the eight screened pathogens were listed in this file. [file 13567_2023_1204_MOESM1_ESM.docx]

**Additional file 1**

| Pathogens | Target genes | Forward primers  (5’-3’) | Reverse primers  (5’-3’) | Ampli-cons  size (bp) | References |
| --- | --- | --- | --- | --- | --- |
| Influenza A virus | M | **M52C:** CTTCTAACCGAGGTCGAAACG | **M253R:** AGGGCATTTTGGACAAAKCGTCTA | 250 | [50] |
| H9 subtype | HA | **H9-Shabat-Fm:** GGAAGAATTAATTATTATTGGTCRGTAC | **H9-Shabat-R:** GCCACCTTTTTCAGTCTGACATT | 185 | [24] |
| NDV | F | **FIP1:** TACTTTGCTCACCCCCCTT | **FIP2:** CATCTTCCCAACTGCCACT | 280 | [51] |
| IBV | UTR | **GU391:** GCTTTTGAGCCTAGCGTT | **GL533:** GCCATGTTGTCACTGTCTATTG | 143 | [52] |
| aMPV | SH | **SH-f:** TAGTTTTGATCTTCCTTGTTGC | **SH-r:** GTAGTTGTGCTCAGCTCTGATA | 200 | [53] |
| ILTV | UL15a | **UL15aF:** TTGCTGTGCTATTTCGCGTG | **UL15aR:** GTAAATCGTTTAGTGCGGCAT | 113 | [54] |
| *MS* | vlhA | **Vlha F:** CCATTGCTCCTGCTGTTAT | **Vlha R:** KMTKCTGTTGTAGTTGCTTCAA | 295 | [55] |
| *MG* | Mgc2 | **Mgc2 F:** CGCAATTTGGTCCTAATCCCCAACA | **Mgc2 R:** TAAACCCACCTCCAGCTTTATTTCC | 300 | [56] |
| *ORT* | *gyrA* | **ORT101F:** TGGGCAAGGGAACTTTGGTT | **ORT101R:** TGTCGGCAAGCATTTCCTCA | 101 | [23] |

aMPV, Avian metapneumovirus; IBV, Infectious Bronchitis virus; ILTV, Infectious Laryngotracheitis virus; MG, *Mycoplasma gallisepticum*; MS, *Mycoplasma synoviae*; NDV, Newcastle Disease virus; ORT, *Ornithobacterium rhinotracheale*.
