# Supplementary material for: Predominance of low pathogenic avian influenza virus H9N2 in the respiratory co-infections in broilers in Tunisia: a longitudinal field study, 2018–2020
Source: Vet Res. 2023 Oct 3;54:88. doi: 10.1186/s13567-023-01204-7 (PMC10548753; doi:10.1186/s13567-023-01204-7)
Supplement: Supplementary file 2 — Additional file 2: Investigated flocks’ information. The date of sampling, the farm and governorate of origin, the size and the age of the investigated flocks were showed in this file. [file 13567_2023_1204_MOESM2_ESM.docx]

**Additional file 2**

| Flocks ID | **Sampling date** | **Governorate** | **Farm** | **Flock size (birds)** | **Age (days)** |
| --- | --- | --- | --- | --- | --- |
| 18-001 | 27/01/18 | BenArous | farm D | 11500 | 15 |
| 18-003 | 15/02/18 | BenArous | farm D | 18400 | 20 |
| 18-004 | 03/04/18 | BenArous | farm B | 9000 | 48 |
| 18-007 | 24/04/18 | BenArous | farm D | 12000 | 28 |
| 18-008 | 26/04/18 | BenArous | farm D | 10200 | 31 |
| 18-010 | 22/05/18 | BenArous | farm F | 11000 | 35 |
| 18-011 | 25/05/18 | BenArous | farm B | 5000 | 27 |
| 18-012 | 31/05/18 | BenArous | farm B | 7000 | 28 |
| 18-013 | 31/05/18 | BenArous | farm B | 7000 | 28 |
| 18-014 | 23/06/18 | Nabeul | farm E | 10000 | 30 |
| 18-015 | 02/07/18 | BenArous | farm F | 12000 | 25 |
| 18-016 | 14/07/18 | BenArous | farm B | 7500 | 18 |
| 18-017 | 01/08/18 | BenArous | farm D | 26000 | 28 |
| 18-019 | 03/08/18 | BenArous | farm B | 7500 | 38 |
| 18-020 | 03/08/18 | BenArous | farm B | 6200 | 36 |
| 18-021 | 13/10/18 | BenArous | farm B | 8000 | 33 |
| 18-022 | 13/10/18 | BenArous | farm B | 8500 | 33 |
| 18-023 | 13/10/18 | BenArous | farm B | 8500 | 33 |
| 18-026 | 31/10/18 | Nabeul | farm E | 10000 | 30 |
| 18-028 | 01/12/18 | BenArous | farm B | 8000 | 28 |
| 18-029 | 01/12/18 | BenArous | farm B | 8500 | 31 |
| 19-035 | 18/02/19 | BenArous | farm D | 16000 | 31 |
| 19-036 | 25/02/19 | BenArous | farm D | 33000 | 35 |
| 19-037 | 04/03/19 | BenArous | farm D | 19500 | 31 |
| 19-038 | 28/03/19 | BenArous | external | 15000 | 30 |
| 19-042 | 22/11/19 | BenArous | farm D | 18000 | 32 |
| 20-047 | 25/02/20 | Manouba | external | 23000 | 15 |
| 20-048 | 25/02/20 | BenArous | farm B | 7000 | 40 |
| 20-049 | 25/02/20 | BenArous | farm B | 6500 | 40 |
| 20-051 | 28/02/20 | BenArous | farm B | 7800 | 39 |
| 20-052 | 04/03/20 | Bizerte | external | 7000 | 28 |
| 20-053 | 10/03/20 | Nabeul | farm E | 8500 | 41 |
| 20-054 | 10/03/20 | Nabeul | farm E | 14500 | 40 |
| 20-055 | 11/03/20 | Bizerte | external | 3700 | 37 |
| 20-056 | 12/03/20 | Nabeul | farm A | 25500 | 50 |
| 20-057 | 16/03/20 | Nabeul | farm C | 18000 | 42 |
| 20-058 | 15/04/20 | BenArous | farm D | 12000 | 47 |
| 20-059 | 05/06/20 | Nabeul | farm C | 16000 | 40 |
| 20-060 | 26/06/20 | Nabeul | external | 7500 | 28 |

**†** Cumulative daily mortality during the first five days of the respiratory outbreak
