# Supplementary material for: Predominance of low pathogenic avian influenza virus H9N2 in the respiratory co-infections in broilers in Tunisia: a longitudinal field study, 2018–2020
Source: Vet Res. 2023 Oct 3;54:88. doi: 10.1186/s13567-023-01204-7 (PMC10548753; doi:10.1186/s13567-023-01204-7)
Supplement: Supplementary file 3 — Additional file 3: The qPCR Ct values of detected pathogens. The Age, the mortality, and the qPCR Ct values of the detected pathogens per flocks were listed in this file. [file 13567_2023_1204_MOESM3_ESM.docx]

**Additinal file 3**

| Pathogen |  |  | **Viral (Ct)^b^** | | | |  | **Bacterial (Ct)** | | |  |
| --- | --- | --- | --- | --- | --- | --- | --- | --- | --- | --- | --- |
| Flocks ID | **Age (day)** | **Mortality^a^ (%)** | **H9N2** | **NDV** | **IBV** | **aMPV** | | **MS** | **MG** | **ORT** | |
| 18-001 | 15 | 1.8 | -^c^ | - | 25.8 | - | | - | - | N.D | |
| 18-003 | 20 | 1 | - | - | 26.05 | - | | 24.9 | - | N.D | |
| 18-004 | 48 | 4 | 25.4 | - | - | - | | 22.5 | - | N.D | |
| 18-007 | 28 | 4.4 | 25.5 | - | - | - | | 27.5 | - | N.D | |
| 18-008 | 31 | 4 | 24.3 | - | - | - | | - | - | N.D | |
| 18-010 | 35 | 8.4 | 22.8 | - | - | - | | - | - | N.D | |
| 18-011 | 27 | 2 | - | - | 21.6 | - | | - | - | N.D | |
| 18-012 | 28 | 3.9 | 34.3 | - | 26.8 | - | | - | - | N.D | |
| 18-013 | 28 | 4.1 | 34.4 | - | 26.1 | - | | - | - | N.D | |
| 18-014 | 30 | 1.6 | 25.3 | - | - | - | | - | - | N.D | |
| 18-015 | 25 | 3.4 | 33.3 | - | 28.0 | - | | - | - | N.D | |
| 18-016 | 18 | 4.2 | - | - | 23.1 | - | | - | - | N.D | |
| 18-017 | 28 | 6.5 | 25.8 | - | 27.1 | - | | - | - | N.D | |
| 18-019 | 38 | 3.1 | 32.1 | - | 27.7 | - | | - | - | N.D | |
| 18-020 | 36 | 20.6 | - | - | 27.6 | - | | - | - | N.D | |
| 18-021 | 33 | 5.4 | - | - | 21.2 | - | | - | - | N.D | |
| 18-022 | 33 | 9.1 | - | - | 25.4 | - | | - | - | N.D | |
| 18-023 | 33 | 1.7 | - | - | 26.9 | - | | - | - | N.D | |
| 18-026 | 30 | 2.4 | - | - | 21.6 | - | | 23.3 | - | N.D | |
| 18-028 | 28 | 1.5 | - | - | 23.4 | - | | - | - | N.D | |
| 18-029 | 31 | 1.6 | - | - | 30.8 | - | | - | - | N.D | |
| 19-035 | 31 | 71.8 | - | 28.9 | - | - | | - | - | N.D | |
| 19-036 | 35 | 6.2 | - | 30 | - | - | | 31.2 | - | N.D | |
| 19-037 | 31 | 61.5 | - | 26.8 | - | - | | - | - | N.D | |
| 19-038 | 30 | 13.2 | - | 28.1 | - | - | | - | - | N.D | |
| 19-042 | 32 | 0.3 | - | - | 19.7 | - | | - | 20.5 | N.D | |
| 20-047 | 15 | 1.7 | - | - | 26.6 | 25.5 | | - | - | N.D | |
| 20-048 | 40 | 3.8 | - | **-** | - | 25.8 | | - | - | 24.3 | |
| 20-049 | 40 | 2.6 | 29.4 | - | - | - | | - | - | 27.6 | |
| 20-051 | 39 | 1.3 | 22.0 | **-** | - | 29.5 | | - | - | 28.7 | |
| 20-052 | 28 | 5 | 34.2 | - | 27.6 | - | | 20.9 | - | 20.1 | |
| 20-053 | 41 | 4.6 | 32.7 | **-** | - | 32.5 | | 32.7 | - | 19.5 | |
| 20-054 | 40 | 1.7 | 34.2 | - | - | 16.4 | | 31.0 | - | 20.7 | |
| 20-055 | 37 | 1.9 | 32.3 | **-** | - | 29.6 | | 32.1 | - | 24.6 | |
| 20-056 | 50 | 4.9 | 32.3 | - | - | 14.9 | | - | - | 20.3 | |
| 20-057 | 42 | 4.2 | 22.4 | **-** | - | 27.1 | | 32.9 | - | 25.9 | |
| 20-058 | 47 | 3 | 34.5 | - | 30.8 | 25.7 | | 31.3 | - | 19.9 | |
| 20-059 | 40 | 10.9 | 28.6 | **-** | - | 28.1 | | 30.4 | - | 23.3 | |
| 20-060 | 28 | 5.6 | 24.6 | **-** | - | 30.2 | | 26.2 | - | 24.5 | |

H9N2, low pathogenic avian influenza virus; aMPV, Avian metapneumovirus; IBV, Infectious Bronchitis virus; ILTV, Infectious Laryngotracheitis virus; MG, *Mycoplasma gallisepticum*; MS, *Mycoplasma synoviae*; NDV, Newcastle Disease virus; ORT, *Ornithobacterium rhinotracheale*

N.D: not done.

^b^ Threshold Cycle.

^a^ Cumulative daily mortality during the first five days of the respiratory outbreak

^c^ Negative
