# Supplementary material for: Predominance of low pathogenic avian influenza virus H9N2 in the respiratory co-infections in broilers in Tunisia: a longitudinal field study, 2018–2020
Source: Vet Res. 2023 Oct 3;54:88. doi: 10.1186/s13567-023-01204-7 (PMC10548753; doi:10.1186/s13567-023-01204-7)
Supplement: Supplementary file 4 — Additional file 4: GenBank accession numbers of nucleotide sequences generated during this study. Virus sequences generated in this study were submitted to GenBank database under the accession numbers listed in this additional file. [file 13567_2023_1204_MOESM4_ESM.docx]

**Additional file 4**

|  | **GenBank accession number(s)** | | | |
| --- | --- | --- | --- | --- |
| Flocks ID | **H9N2** | **NDV** | **IBV** | **aMPV** |
| 18-001 |  |  | OQ199502 |  |
| 18-004 | OQ179925 |  |  |  |
| 18-008 | OQ179926 |  |  |  |
| 18-010 | OQ179927 |  |  |  |
| 18-011 |  |  | OQ199501 |  |
| 18-013 |  |  | OQ199500 |  |
| 18-014 | OQ179928 |  |  |  |
| 18-016 |  |  | OQ199499 |  |
| 18-017 | OQ179929 |  |  |  |
| 18-019 |  |  | OQ199498 |  |
| 18-020 |  |  | OQ199507 |  |
| 18-021 |  |  | OQ199497 |  |
| 18-022 |  |  | OQ199496 |  |
| 18-023 |  |  | OQ199495 |  |
| 18-026 |  |  | OQ199494 |  |
| 19-037 |  | OQ199524 |  |  |
| 19-038 |  | OQ199525 |  |  |
| 19-042 |  |  | OQ199504 |  |
| 20-047 |  |  | OQ199506 |  |
| 20-048 |  |  |  | OQ199508 |
| 20-051 |  |  |  | OQ199509 |
| 20-052 |  |  | OQ199503 |  |
| 20-054 |  |  |  | OQ199510 |
| 20-055 |  |  |  | OQ199511 |
| 20-056 |  |  |  | OQ199512 |
| 20-057 | OQ179924 |  |  |  |
| 20-058 |  |  | OQ199505 | OQ199513 |
| 20-059 | OQ179930 |  |  | OQ199514 |
| 20-060 |  |  | OQ199502 | OQ199515 |
